# Replicability of Ultrasonic Molding for Processing Thin-Wall Polystyrene Plates with a Microchannel

**DOI:** 10.3390/ma11081320

**Published:** 2018-07-30

**Authors:** I. Ferrer, M. Vives-Mestres, A. Manresa, M. L. Garcia-Romeu

**Affiliations:** 1 Department of Mechanical Engineering & Industrial Construction, University of Girona, 17004 Girona, Spain; manresa.ariadna@gmail.com (A.M.); mluisa.gromeu@udg.edu (M.L.G.-R.); 2 Department of Computer Science, Applied Mathematics & Statistics, University of Girona, 17004 Girona, Spain; marina.vives@imae.udg.edu

**Keywords:** ultrasonic molding, microchannel, thin-wall plate, replicability, polystyrene, medical devices

## Abstract

Ultrasonic molding is a new technology for processing small and micro polymeric components with reasonable cost and energy savings when small and medium batch sizes are required. However, when microcomponents are manufactured, the replicability of different micro features has to be guaranteed. The aim is to investigate the capability of ultrasonic molding technology for processing thin-wall plates of polystyrene with a microchannel, analyzing the filling behavior, the optical transparency, and the dimensional accuracy of the thin plate. The replicability of the manufactured microchannel is studied according to dimension and shape. The results reveal that plunger velocity influences transparency and filling cavity, whereas the vibration amplitude has less effect in both cases. The thickness deviation achieved on the final part is below 7% and the replication of the microchannel is better in depth than width, obtaining an average deviation of 4% and 11%, respectively. This replication also depends on the orientation of the microchannels and the distance from the injection gate. The replicability and repeatability for processing thin-wall plates with microchannel in polystyrene polymer are proved in this paper.

## 1. Introduction

In recent years, the use of polymer materials in industry has increased notably, with sectors such as micro-electromechanical systems (MEMS), aeronautics, and biomedicine slightly influenced by this trend. Some reasons for this strong tendency include the reduced weight of the component, the increment of the complexity of 3D geometries, the trend toward miniaturization of parts, the increment of functionality of products, the high performance requirement of the materials, and the emergence of many new polymers with very different characteristics [1,2].

Miniaturization of mechanical systems and devices leads to the development of microcomponents (such as microgears, microfluidic devices, and micro-optical lenses), small parts with microfeatures of high aspect ratio, or parts having microfeatures pattered on the surface. When processing these miniature polymeric components, injection and microinjection molding are the commonly used manufacturing techniques because of their high productivity, cost-effectiveness, high replicability and repeatability, high capability for producing an extended variety of polymeric components, tight tolerances, and complex shapes [1,3].

However, the replicability of thermoplastic components at the micro scale is quite complex, and there are several parameters that influence the process, such as injection speed, injection pressure, melting temperature, mold temperature, and others [4]. Actually, there are strong interactions among process parameters, process variations, mold design and configuration, part shrinkage, and internal material stress that reduce process repeatability and replicability [4]. Consequently, new technologies are being researched, such as compression molding [4,5], vacuum injection molding [4], ultrasonic injection molding [6,7], ultra-high-speed injection molding [8,9], and 3D printing [10].

Focusing on these molding manufacturing processes, in recent years, several researchers have studied the influence of process parameters to obtain microgeometries with high aspect ratio using different polymeric materials. Attia and Alcock [11] evaluated the weight of five products with microscaled features of polymethylmethacrylate (PMMA) manufactured by microinjection molding and found that the more relevant parameter to increase product weight was the holding pressure, but they also found that when complex geometries are molded, injection speed and mold temperature also become significant. Chen et al. [12] replicated a microfluidic chip by microinjection molding using a plate of 80 mm length, 40 mm width, and 0.2 mm thickness with microchannels of 100 µm width and 30 µm depth. They tested different processing parameters for manufacturing four polymer resins, cyclic olefin copolymer (COC), polystyrene (PS), polycarbonate (PC), and polymethylmethacrylate (PMMA), and found that, in general, the microchannels manufactured in COC and PS achieved the required accuracy, with higher dimensional accuracy when COC resin was used. Moreover, they found that microchannel width and depth improved when higher temperatures, for both molding and melting, higher injection speeds, and higher packing pressures were used. Lucchetta et al. [13] replicated a circular microfilter of PS that included squared ribs of 150 × 150 µm features separated by 50 μm. They focused on analyzing the influence of the mold material with different thermal diffusivity and mold temperature on part filling, without measuring in detail the microfeature dimensions achieved. As a result, ceramic mold material seems to replicate better than metallic due to its capability to delay polymer skin solidification when the cavity is being filled. Masato et al. [14] studied the replication of micro holes (4 µm in diameter and 5 µm in depth) regarding the thickness variation of the molded part of PS material. Their results revealed that part thickness and mold temperature influence replication. The replication accuracy improved for higher mold temperature and thinner part thickness, because the pressure of the melt inside the mold increased and the polymer could reach further. Mahmoodi et al. [2] investigated the filling behavior and dimensional accuracy of a thin part (6 × 6 mm square, 200 µm thick) using microinjection molding and adding chemical blowing agents and wood fibers. Injection pressure and mold temperature were modified in both experiments and the numerical model obtained consistent results. The higher the mold temperature and pressure were, the better the filling ratio achieved. Their results also revealed that the in-flow shrinkage percentage became slightly higher than the cross-flow shrinkage percentage. Sortino et al. [4] evaluated the replicability of conventional injection molding, injection compression molding (ICM), and vacuum injection molding (VIM) when manufacturing micro-optical devices with prism patterns using PMMA. They found the holding pressure to be the most influential parameter in the injection molding process, then the injection pressure, and injection speed the least influential. In this regard, the ICM process resulted in the most precise and best feature replication, and VIM the worst. Sato et al. [6] studied the application of ultrasonic injection molding (UIM) when replicating optical lens surfaces. What makes that process different from USM is that ultrasonic energy is applied to the melted material during the mold cavity filling. They varied the oscillation time and obtained the best results in replication when the ultrasonic energy was applied at end of the injection process. This energy locally increases the polymer temperature and causes an oscillatory polymer flow that improves replicability and avoids shrinkage during the packaging and cooling stages.

**Figure 1:**
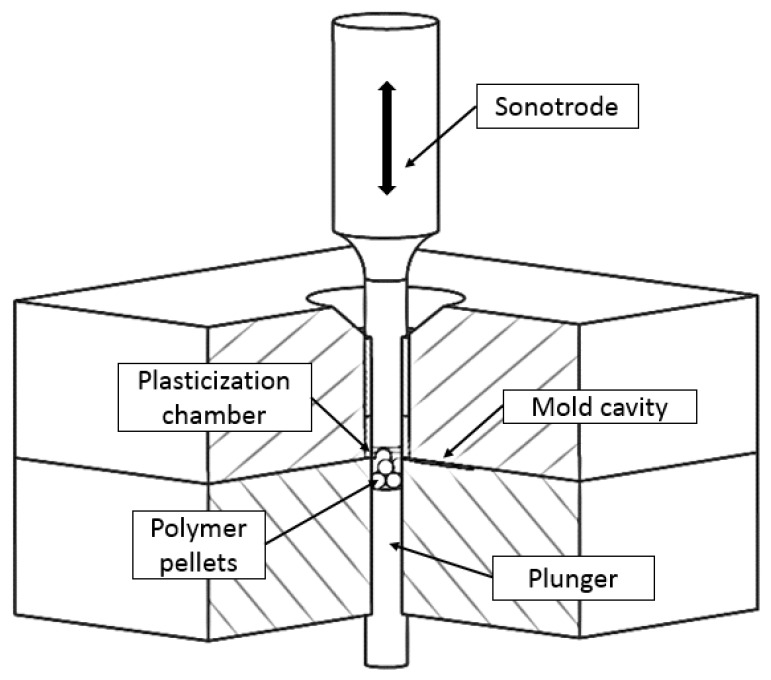
Ultrasonic molding process schema.

Ultrasonic molding is a new manufacturing technology to obtain micropolymeric parts. In ultrasonic molding, the raw material is melted by the direct application of ultrasonic energy. In this process, first the pellets are located in plasticization chamber of the mold (Figure 1), then the sonotrode moves into the mold, vibrates, and transmits the vibrational energy to the material. Then, while the sonotrode is vibrating and the material is being melted, the plunger moves, pushing the melted material into the mold cavity. When the plunger reaches the final position, the mold cavity is filled and it starts the cooling and packing stages. After applying the compaction forces and once the material solidifies, the sonotrode returns to the initial position and the mold can be opened to extract the final part.

Each molding cycle only requires the amount of material to fill the mold cavity and the plasticizer chamber, thus no extra material is melted into the barrel before the process, as in conventional or microinjection molding. This represents an important material and energy savings for small-batch production and high-cost materials (i.e., implantable grade or reinforced materials). Moreover, the process is quite flexible for testing different materials and geometries, because all the material placed into the mold is removed after each cycle. The vibrational energy applied directly to the material produces high heating rates, which allows fast polymer plasticization [15]. Thus, the material is kept at higher temperatures during shorter periods of time, typically less than 5 s, reducing the residence time in comparison to conventional injection molding, and therefore material degradation can be avoided [7].

The ultrasonic vibration is applied by means of the acoustic unit, which is composed of the generator, the transducer, the booster, and the sonotrode. The transducer converts the electrical energy from the generator into vibrations, which can be amplified or reduced by the booster, and the sonotrode is the final element that transmits the vibrational amplitude to the material. The sonotrode is a mechanical element that has to be designed to vibrate in a longitudinal mode at the operating frequency of the acoustic unit, and with the required tolerances to fulfill the clearance requirements with the plasticization chamber dimensions.

There are a few published studies regarding this novel technology dealing with the influence of the processing conditions on manufacturing components. Michaeli et al. [16] can be considered the pioneers of this technology. Through several research works, they proved that ultrasonic energy is capable of melting small amounts of thermoplastic polymer for molded parts. Moreover, they verified the integrity of the polymer after being processed in two ways: internal homogeneity and physical properties, such as weight [15,16,17,18]. They processed PC [15], polyoxymethylene (POM), and polypropylene (PP) [19], and in all of the cases simple geometries were used, such as cylinders or plates. Polymer plasticization occurs mainly by two mechanisms, internal material heating and friction between pellets [19]. In this regard, the melt temperature varies as a function of different processing parameters, such as vibration amplitude and vibration frequency, the pressure applied to the material, or the material properties of the pellets [18]. In recent studies, the influence of processing conditions on the fabrication of different polymeric components has been analyzed. Sacristán et al. [20] studied the influence of vibration amplitude and pressure on polymer properties. They manufactured mini tensile test specimens (1.5 × 0.1 × 0.1 cm^3^) of poly (lactic acid) (PLA) and noted that the cavity filling improved when high amplitude and lower pressure were applied. However, polymer degradation and material homogeneity had the opposite behavior. When higher levels of vibration amplitude and pressure were used, the polymer degraded, whereas using the lowest levels achieved better polymer homogeneity. Later, Planellas et al. [21] used the same geometry to test the feasibility of ultrasonic molding for processing polylactide (PLA) and polybutylene succinate (PBS). Although both polymers were manufactured without molecular degradation, optimization of the processing conditions would be required to obtain better results on the final part. The introduction of clay silicate particles in the polymers did not influence PLA properties but affected PBS degradation. In both cases, structures with high orientation of clay nanosheets were oriented in the direction of polymer flow.

Actually, the literature reveals that there is strong interest in studying the influence of processing conditions on several precision molding processes of microfeature replicability in recent years. However, so far, there are only a few studies about the manufacturability of microfeatures using ultrasonic molding technology. Consequently, the aim in this research is to investigate the process capability of ultrasonic molding for processing thin-plate components with a microchannel of high aspect ratio using PS polymer. Specimens were manufactured under different conditions of vibration amplitudes and injection velocities to evaluate the filling behavior, the optical transparency, and the dimensional accuracy in the plate and the microchannel. The replicability of the manufactured microchannel was studied from the point of view of dimension and shape. The material was used for its good replicability and optical properties, which are required features when manufacturing microfluidic components.

## 2. Materials and Methods

### 2.1. Part Geometry, Materials, and Mold

**Figure 2:**
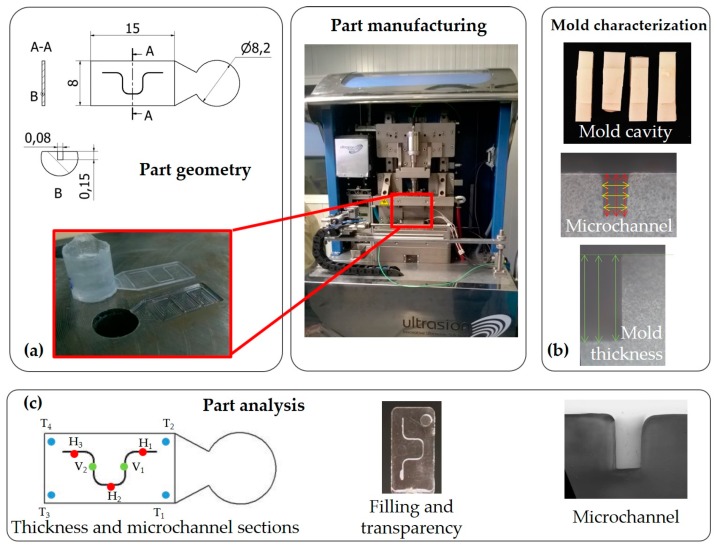
Method and experimental setup: (**a**) part geometry; (**b**) mold characterization; and (**c**) part analysis.

The test geometry used in this experiment was a thin-walled rectangular specimen of 15 mm × 8 mm × 0.55 mm with a direct fan gate (Figure 2a). The plate contained a microchannel of 150 µm depth and 80 µm width and had both parallel and perpendicular zones to flow direction at different distances from the material entry. 

Transparent STYRON™ 678E PS resin was used for the experiment. This material is characterized by its optical clarity, transparency, biocompatibility, low water absorption, high flow ability, and high impact strength compared to other polymers such as silicon or glass.

An experimental mold of steel with the cavity on the lower half was used to replicate the geometry and obtain the final product. It included an ejector to facilitate the expulsion of the part, making a circular mark in final part (Figure 2c). It was manufactured using a Deckel-Maho 64 V Linear and the plasticization chamber was allocated at the mold upper half by a cavity of Ø 8.2 mm. The mold was characterized after being manufactured to guarantee repeatable measurements of the microchannels (Figure 2b). A silicon pattern was created to measure it. The thickness was measured in each corner of the specimen (T_1_ Mold, T_2_ Mold, T_3_ Mold, and T_4_ Mold) by a digital micrometer (Micromar 40 EWV). The microchannel dimensions were measured in 5 sections (Figure 2c), 3 in the inflow direction (H_1_ Mold, H_2_ Mold, and H_3_ Mold) and 2 in the cross-flow direction (V_1_ Mold and V_2_ Mold) by a Leica DMR-XA optical microscope (Meyer Instruments Inc., Houston, TX, USA). Three measurements were taken of each section and thickness (see mean results in Table 1). The results reveal that the thickness of the manufactured mold was 14.3% bigger than the theoretical one, whereas the microchannel dimensions were 6.6% smaller in depth and 10% in width. 

According to the mold dimensions and considering the extra amount of material in the plasticizer chamber, around 300 mg of material was provided in each experiment. 

### 2.2. Part Analysis

The specimens were manufactured using a Sonorus 1G Ultrasound Micro Molding Machine. The responses analyzed were part filling, transparency, thickness, and microchannel formation in 5 sections of the microchannel path (Figure 2c).

- Part filling was assessed by a ratio that compares the obtained part length with the expected theoretical length. Despite only complete parts being valid, corresponding to 100% filling, the influence of the processing parameters on the percentage of filling was also evaluated.
- Part transparency was evaluated visually and categorized in a qualitative scale divided into 7 levels and transmittance values (Table 2). Levels 1–4 represent blurred parts classified according to the percentage of cloudy zones, and levels 5–7 are transparent parts ordered regarding the presence of defects. Blurred parts could be caused by the presence of nonmelted material, weld lines, or polymer degradation, whereas defects include bubbles, sink marks, or burrs. Moreover, transmittance values according to this qualitative scale were measured by a UV-2401PC Shimadzu spectrometer using wavelengths between 400 and 600 nm. Six specimens were taken to measure each category and the mean was calculated.
- Part thickness was measured in the same corners as mold thickness (Figure 2c), although in this case the variables are referred to as T_1_ Part, T_2_ Part, T_3_ Part, and T_4_ Part. A Micromar 40 EWV digital micrometer was used to measure them.
- Microchannel formation was assessed by dimensions (width and depth) and shape. Five sections of the microchannel path were analyzed in each specimen and 3 specimens were selected for each combination of process parameter, on the condition of complete parts. The selected specimens were cut in each section (in-flow and cross-flow direction), embedded into 40 mm of Axson RSF816 epoxy resin, prepolished by Struers Knuth Rotor-3 equipment and finished by a Buehler Ecomet sander polishing with alumina powder of 1, 3, and 9.5 microns. The observation of the microchannel was done using a Leica DMR-XA optical microscope with 5×, 10×, and 20× Plan-Apochromat lenses. The microchannel dimensions were measured by Fiji software and 3 measures of depth and width were taken for each specimen.

### 2.3. Experimental Design and Procedure

In this research, a sequential design of experiments was performed. That is, start with simple designs and few experiments (Phase 1), analyze them, and, if necessary, proceed with further experiments to conclude with the relationship between the process parameters and the part responses (Phase 2). This methodology is known as response surface analysis [22] and it allows selection of the best manufacturing conditions to achieve the part requirements [23]. In this research, two process parameters were varied: plunger velocity (V) and amplitude (A) (also referred as factors of interest).

However, before starting the sequential design of experiments, a preliminary screening was carried out to limit the parameter values to guarantee a filling cavity and a level of transparency acceptable to the polymer application. Velocity was varied from 1 to 6 mm/s and amplitude from 10 to 90 µm. The results revealed that the filling cavity improved when velocity decreased, whereas amplitude did not have much effect. Otherwise, visual transparency improved when velocity increased and when low amplitudes were used. Consequently, the process parameters in the design of experiments (DOE) were limited to amplitude range 14.06–70.31 µm and velocity 1–4 m/s. 

**Figure 3:**
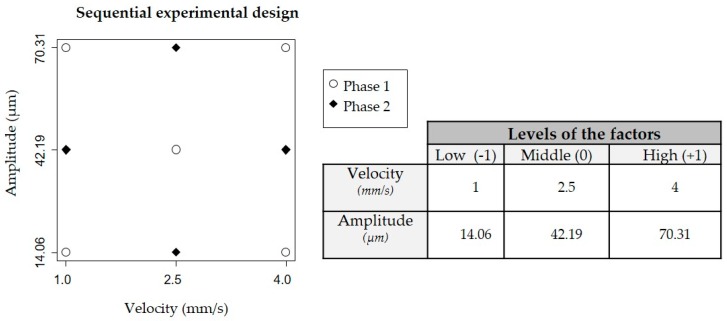
Sequential experimental design: Phase 1 (2^k^ with a central point) and Phase 2 (3^k^); k = 2.

Phase 1 started deploying a two-level factorial design (2^k^, where k = 2) with a central point (2^k^ + CP). Figure 3 shows the 5 experimental runs represented by ◯. For each combination of process parameters 13 parts were manufactured; the first 3 were discarded to stabilize the process and the other 10 were analyzed. Each experimental run was replicated 3 times in completely randomized order. The central point runs allow for checking the trend in the middle point of the process parameters values. When this trend is a curve, more experiments should be done to estimate the quadratic effect, which was done in Phase 2. Phase 2 went on with a 3-level factorial design (3^k^), which was an extension of the previous design (2^k^ + CP). Here, 4 new runs were performed (◆ in Figure 3) following the same pattern of 10 replicates and 3 repeats. 

A set of process parameters was kept constant during both experimental phases. Mold temperature was set according to a raw material datasheet for injection molding applications (50 °C), cooling time was 10 s, with a packing force of 5000 N. Material dosage was approximately 0.3 g and vibration frequency was 30,000 Hz. Regarding the injection strategy selected, the plunger pushes the materials against the sonotrode, which is fixed in a position close to the mold cavity beginning. Its vibration melts the material and the plunger force pushes the melting material into the mold cavity. 

The full model was estimated in each phase and for each response, including first-order and interactions in both phases and the quadratic terms in Phase 2. Then, the nonsignificant terms (one at each step) were sequentially removed based on the tests of individual regression coefficients. Each model was analyzed in terms of the fit statistics: R^2^-adjusted and root mean square error (RMSE). The model shows a good fit if the R^2^ statistic is high and RMSE is low. For each model, the test for significance of regression (*p*-value associated with the model in an analysis of variance, ANOVA) was observed. Analyzing the *p*-value resulting from ANOVA, *p*-value < α (α is the significance level) indicates that the regression is significant. The last step before validating the model is to check adequacy. It allows examination of whether the fitted model offers an adequate approximation to the real system and corroboration that the least squares regression assumptions are met, that is, that residuals are normal independent and identically distributed N (0, σ2). The first is checked by plotting observed values on the model response surface and the second by looking at different model diagnostic graphs. In the present paper, the residual vs. predicted values graph was used to check for independency and homoscedasticity and the normal probability plot was used to check normality. Conclusions on graphical analysis can be confirmed with tests such as the Shapiro–Wilk normality test or the test on correlation coefficient. 

Finally, the retained model is plotted by a response surface to enhance interpretability and identify the process conditions where the responses become optimal. R software [24] was used to carry out the statistical analysis, and the significance level in all cases is α = 0.1.

## 3. Results and Discussion

Table 3 presents the average results for the percentage of completed specimens, the transparency achieved, and the average thickness for each combination of processing parameters. There is no thickness dimension when any completed part was achieved.

### 3.1. Transparency

**Figure 4:**
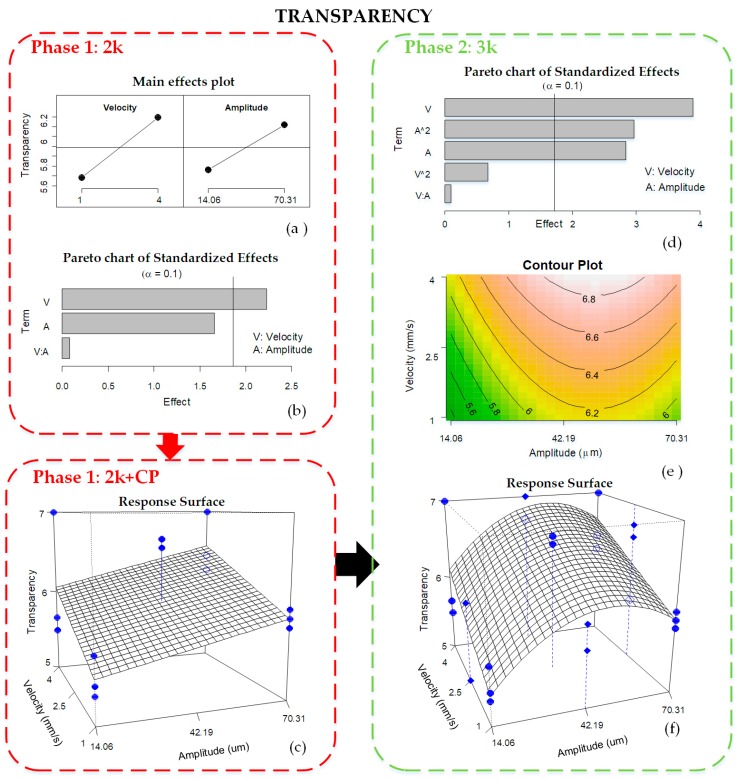
Analysis of transparency results: (**a**) main effects plot 2^k^; (**b**) Pareto chart of effects 2^k^; (**c**) response surface 2^k^ + CP; (**d**) response surface 3^k^; (**e**) Pareto chart of standardized effects 3^k^; and (**f**) contour plot of final 3^k^ model; k = 2.

First, the transparency results of the Phase 1 2^k^ + CP experimental runs are analyzed, although initially the central point is excluded. Figure 4a shows the main effects plot of the response mean for each level of the process parameters. It reveals that transparency is better when both velocity and amplitude levels are higher. Besides, the Pareto chart of the standardized effects (Figure 4b) reflects those factors, which contribute significantly to explain transparency. Really, both velocity and amplitude affect transparency, although amplitude is a near-significant factor. On the contrary, the interaction between the two is not significant, so it is excluded from the model and re-estimated. In the final model, the resulting regression is significant, and both velocity and amplitude become significant parameters. The residuals of that model are normal (Shapiro–Wilk normality test *p*-value = 0.7574) and homoscedastic, and the model is able to explain almost 50% of the variability of the response (R^2^adj = 0.49) and has RMSE = 0.45.

Figure 4c shows the response surface of Phase 1 including the experimental results of the central point experiments (2^k^ + CP). Three replicates per experimental combination resulting from the mean of 10 repeats are represented by dots. In this graph, the responses at the central point are far from the surface, which means a lack of linearity between low and high factor levels and the possibility of a curve trend. Indeed, the test on the existence of a curve effect was significant, therefore more experiments were run to estimate this quadratic effect (as introduced in Section 2.3). Consequently, four new experiments with three replicates were performed and analyzed (Figure 3; Phase 2, 3^k^).

Taking all the experiments from Phase 2, the factors influencing transparency are velocity (V) and amplitude (A and A2) (Figure 4d). The model is re-estimated after suppressing the nonsignificant terms and the resulting response surface is represented in Figure 4f. In this figure, the response surface is curved along the amplitude factor and is quite close to the experimental results (●), which is reflected by lower RMSE of 0.39. This model is able to explain 60% of the variability of transparency (R2adj = 0.6), which is a better result than the previous one (Phase 1, 2^k^). Residuals are normally distributed (Shapiro–Wilk normality test *p*-value = 0.7672) and homoscedastic. The contour plot of the retained model is shown in Figure 4e. This process map shows the manufacturing conditions in which part transparency becomes better. It is at high velocity of 4 mm/s and amplitude around 42.19 μm.

High plunger velocity fills the mold cavity quickly, reducing the contact time of melting into the mold cavity and dropping the melt temperature, the same as the injection speed in microinjection molding [10]. Otherwise, low velocity increases the contact time of the polymer with ultrasonic energy, increasing the melting temperature and cooling time. As a result, degradation, internal cracking, and bubbles appear in the final part, notably reducing the transparency.

**Figure 5:**
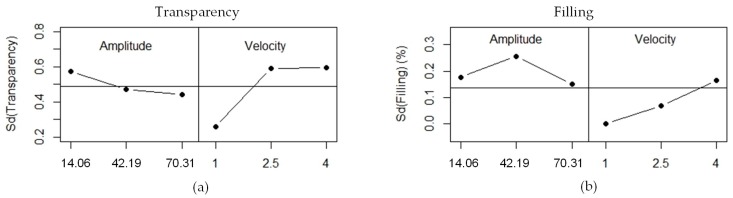
Standard deviation analysis: (**a**) transparency; and (**b**) part filling.

Analyzing the transparency variability of the three replicates (Figure 5a), it is observed that the dispersion is really low. Maximum variability of SD (0.6) is achieved. For low velocity values, transparency repeatability is better, whereas across amplitude, the standard deviation is not greatly affected.

### 3.2. Filling

As shown in Table 3, most fillings are above 50% and even between 80% and 100%, therefore highly biased to the highest levels. Although it was expected, due to the range of process parameters adjusted during the preliminary screening, this response variable had to be transformed to accomplish the regression assumptions. A logit transformation was applied. In our case, we decided to logit transform the variable logit (Filling) = log(Filling/(100 − Filling) and adjust the model to this new response variable. When filling is 100%, its logit cannot be computed, and after some trials, we decided to replace those cases by 10.

The filling ratio analysis was done directly, taking the results from Phase 2, because the quadratic response surface estimated to explain transparency was shown to be appropriate, and measuring it is not expensive or time-consuming.

**Figure 6:**
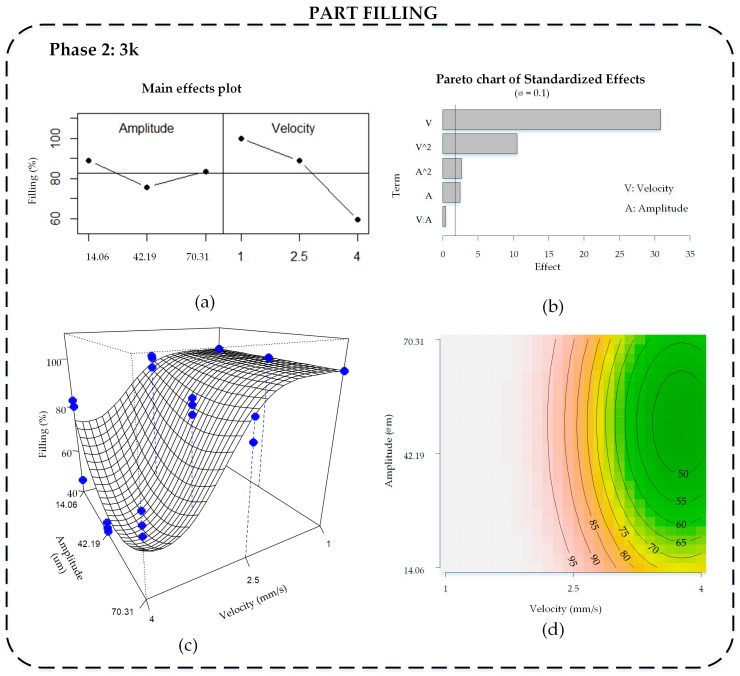
Analysis of cavity filling results: (**a**) main effects plot, 3^k^; (**b**) Pareto chart of standardized effects, 3^k^; (**c**) response surface, 3^k^; and (**d**) contour plot of final 3^k^ model, k = 2.

The main effects plot for filling is shown in Figure 6a and the Pareto chart of standardized effects in Figure 6b. Here, velocity is the factor that most contributes to the filling ratio. When velocity is low (1 mm/s), filling is highest (100%). On the other hand, changes in amplitude do not greatly change the filling; however, as the Pareto diagram shows, this small change becomes significant. 

The model that better explains part filling includes velocity and amplitude factors, with both linear and quadratic terms, and without interaction effect. It explains 97.7% of the data variability (R2adj = 0.977), with normally distributed residuals (Shapiro–Wilk normality test *p*-value = 0.6944) and homoscedasticity.

Figure 6c illustrates the response surface including the experimental results (●), and Figure 6d shows the contour plot of the same model. In both plots, the responses are represented in original scale. It can be seen how the logit transformation enables a response surface limited to 100%, which could not be attained using the original filling variable. Maximum filling is attained at low velocity levels for any amplitude values, getting mostly completed parts. Actually, when velocity is lower, melting temperature increases due to more ultrasonic energy being applied, and complete filling is guaranteed [7,25]. The polymer temperature drops more slowly and it has more time to fill the mold cavity properly. At high velocity, the influence of amplitude is higher, due to the filling reaching the minimum value at the middle value, and improves when it gets away, although the majority are below 70% of filling. During the experiment, when both amplitude and velocity were at the highest values, the parts became too breakable due to the high level of energy that the polymer received, and often broke or seemed to be degraded. 

The standard deviation of the filling is also very low, because less than 0.3% of the variability is achieved (Figure 5b). It can be seen that an increment of velocity is associated with an increment of the standard deviation of filling, which means that the experiments replicate less. Indeed, at a velocity of 1 mm/s, the standard deviation is 0 because all three replicates have the same 100% filling result. Amplitude does not greatly affect the standard deviation.

Comparing filling and transparency response, we observe that is not possible to find the manufacturing conditions that maximize both responses. High velocity and medium amplitude values improve the transparency but worsen the filling cavity. Thus, depending on the application, the manufacturing conditions should be adapted.

### 3.3. Part Thickness

Part thickness was only measured in completely filled parts (Table 3 shows average results) and the thickness difference (D) was calculated to evaluate this response. The thickness difference (D) is the result for comparing part thickness measured in each corner of the parts (T_i_ Part, i = 1…4) regarding the real mold thickness measured in the same corresponding corner (T_1_ Mold, i = 1…4). Thus D_i_ = T_i_ Part—T_i_ Mold, i = 1…4, resulting in four variables for each part. On the one hand, a positive value of D_i_ indicates that the part is thicker than the real mold thickness. On the other hand, a negative value of D_i_ indicates that the piece is thinner than the real mold thickness. 

**Figure 7:**
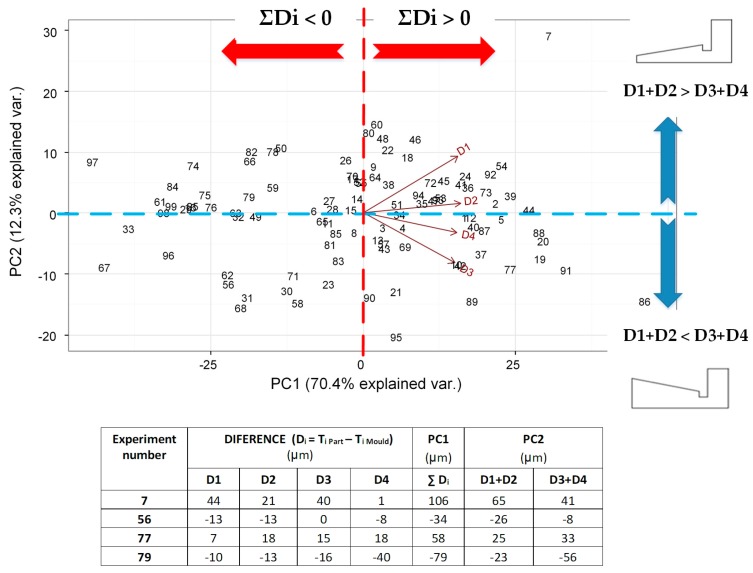
Biplot resulting from principal component analysis of Di, where D_i_ = T_i_ Part—T_i_ Mold, i = 1…4 measured at four different part points.

The objective here is to analyze those four variables and relate them to the manufacturing factors (amplitude and velocity). Nevertheless, as a four-dimensional space cannot be plotted, they were represented in a lower-dimensional space by means of principal component analysis (PCA). PCA is a statistical technique used to reduce the number of variables to represent the dimensions that have more variability in the results. In this case, the four variables (Di, i = 1…4) are represented in a biplot that retains almost 83% of the variability of the dataset (Figure 7). Each number plotted in this graph is a vector that contains the four differences (Di) measured in each complete part (see numerical examples in Figure 7). In Figure 7, the first two principal components (PC1 and PC2) are represented. 

The first component (PC1) in the horizontal axis is interpreted as the sum of all thickness differences, that is, PC1 ≈ ΣDi. Thus, parts that are on the right-hand side of the graph have positive ΣDi values, meaning that, in general, the part thickness is thicker than the mold (see values of experiments 7 and 77 in Figure 7). On the contrary, the parts that are on the left of the graph are narrower than the mold (see experiments 56 and 79). This component (PC1) is the most important one, because it explains 70.4% of variability. 

Actually, the expected result would be that most of the pieces were equal to or thinner than the mold thickness, due to the shrinkage effect that commonly happens in injection molding processes, such as microinjection and conventional injection molding, although, as is known, amorphous polymers are less affected by this. However, the experimental results in this research determined by ultrasonic molding technology reveal that around 50% of the obtained parts had an opposite trend to what was expected and seemed to be affected by a releasement phenomenon. The main hypothesis to explain this is that the polymer was still too warm when the mold opened and the pressure released. Thus, the polymer relieved its stored internal energy (both thermal and ultrasonic) in the easier direction, lightly expanding the parts, similar to the swelling behavior in extrusion processes. Possibly the cooling time used in the experiment was not enough to guarantee final cooling of the polymer, although the parts seemed complete when the mold opened. This cooling time was taken from the microinjection molding process; however, these results reveal that the melting temperature in ultrasonic molding becomes higher than in microinjection molding. 

Despite this effect, the average of PC1 ≈ Σ D_i_ is around 50 µm, which represents less than 7% of thickness dimensional error, which is acceptable for many applications. 

The second component (PC2) in the vertical direction axis is interpreted as D1 + D2 against D3 + D4. It lets us compare the thickness of the corners nearest to the injection entry those farthest. When the parts have positive values of PC2 (upper side of the graph), this means that they have larger values of D1 and D2 than D3 and D4, meaning that the part is thicker on the side of the injection flow (see experiments 7 and 79). On the other hand, the parts on the lower side of the graph have lower D1 and D2 values and higher D3 and D4 values, which means that the part is thicker at the farthest side of the injection entry (see experiments 77 and 56). Note that variables D1 and D2 are grouped together, as they are closely associated or correlated from a statistical point of view, meaning that parts with high (low) values of D1 also have high (low) values of D2. The same applies to D3 and D4. 

**Figure 8:**
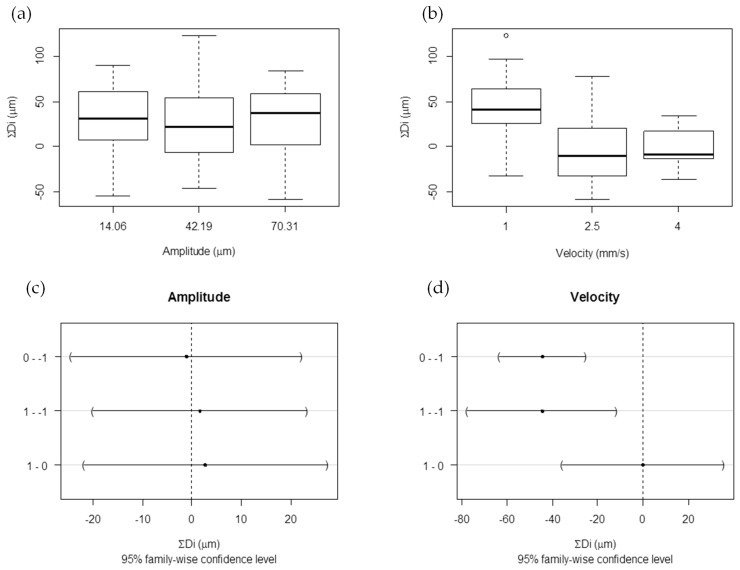
Multiple box plots: (**a**) amplitude and (**b**) velocity and, ANOVA analysis: (**c**) amplitude and (**d**) velocity for first principal component (PC1 = ΣDi).

Once the characterization of part thickness was explained, it was interesting to relate those characteristics with velocity and amplitude factors. Actually, the first principal component (PC1 = ΣDi) showed more significant differences between factor levels, therefore it was analyzed. Taking PC1 results, a graphical analysis by multiple box plots (Figure 8a,b) and numerical ANOVA (Figure 8c,d) were done, comparing the sum of all difference thicknesses (ΣDi) for the different factor levels.

Regarding velocity, when it is low (1 mm/s), the parts are, on average, thicker than the mold, whereas when velocity is at a medium or high level, the parts are, on average, thinner than the mold. Considering amplitude, the parts are, on average, thicker than the mold. As with transparency and the part filling response, amplitude does not really influence part thickness, but velocity does. ANOVA enables us to test the hypothesis that the means of the three levels are equal. In the case of amplitude, the averages are equals, and, for velocity, the medium and highest level are equal. 

When the process is slow (velocity 1 mm/s), the ultrasonic time is longer and the material achieves higher temperatures. Consequently, when the mold is opened, the part temperature is too hot and the polymer releases its internal energy, leading to slightly increased thickness. 

### 3.4. Microchannel Results

The microchannels were studied in three sections in the in-flow direction (H_1_, H_2_, and H_3_) and two in the cross-flow direction (V_1_ and V_2_). Three completed parts for each combination of process parameters were selected, resulting in 15 sections for each combination of process parameters. Three measures of depth and width of each microchannel were taken in each section, similar to the mold characterization shown in Figure 2b. Altogether, 630 measures were collected, and the average values were used to evaluate these results. 

Three issues of the microchannel results are assessed. The first is the influence of the process parameters on the width and depth of the microchannel independently of section location. The second is an examination of these dimensions according the section location (H_1_, H_2_, H_3,_ V_1_, and V_2_). Finally, the microchannel shape is observed and discussed in detail. 

**Figure 9:**
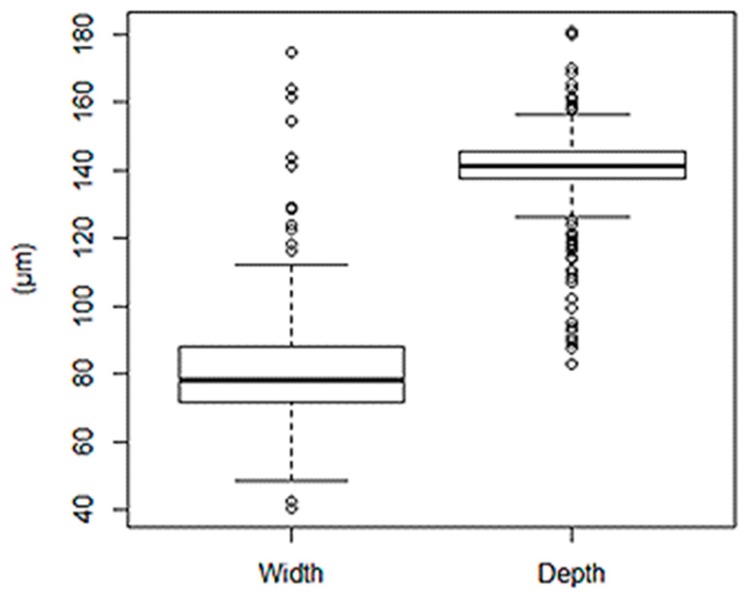
Multiple box plot of the microchannel.

The average microchannel dimension is around 139 µm in depth and 81 μm in width, resulting in 1 µm smaller than the mold depth (140 µm), 9 µm bigger than the mold width (72 µm), and a low variability, as shown in Figure 9. It is an error of 1% and 12%, respectively. Thus, polystyrene has good replicability when microfeatures are manufactured by ultrasonic molding technology. According to Chakraborty et al. [10], the replicability is better in depth than in width.

**Figure 10:**
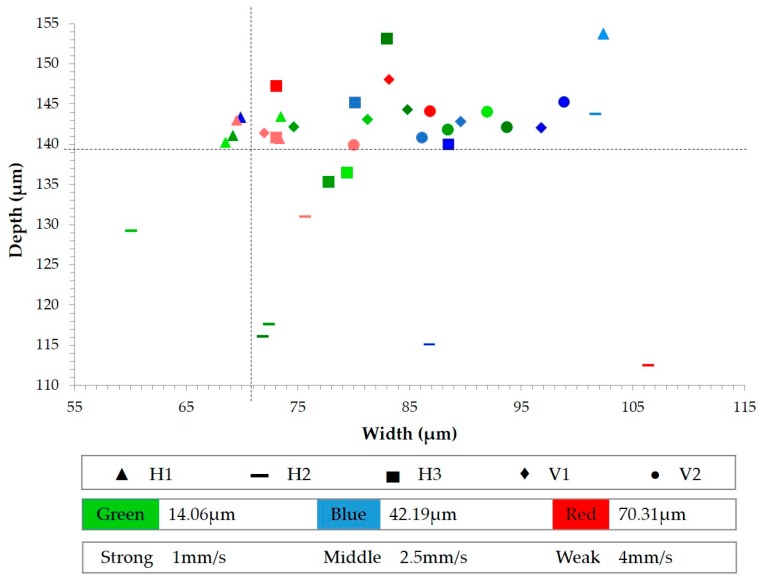
Microchannel dimensions.

ANOVA reveals that the dimensions of the microchannel are not affected by velocity, independently of section location. However, amplitude does slightly affect the width dimension. In contrast, the section location (H_1_, H_2_, H_3_, V_1_, and V_2_) is the factor that contributes most to microchannel replicability (Figure 10). This figure plots the averages of microchannel dimensions, specifying section location (by the shape of the label), amplitude (by color), and velocity (by the color intensity). It reveals that the depth results are nearer the mold dimensions and less dispersed than the width results, just as in [26] with molded PMMA microchannels, where they obtained good replication in depth but poor in width. On average, the replication accuracy for depth is less than 4% and for width is less than 11%, the same as achieved by Chakraborty et al. [10]. Focusing on in-flow sections, the H_1_ dimensions (▲) are very close to the target values for both depth and width. Basically, proximity to the injection entry supports this result [14]. The H_2_ results (▬) are the worst, since both depth and width are very far away from the target values. The H_3_ dimensions (▄) are between H_1_ and H_2_ results. Analyzing the cross-flow sections, V_1_ (♦) and V_2_ (●), the results are grouped more around the target depth than the target width. Without considering H_2_ results, it is observed that the results of cross-flow sections are more dispersed than in-flow ones. The deviated results of H_2_ can be due to a lack of homogeneity in the mold temperature and the proximity of this section to the hottest mold zone. For this reason, the polymer of the H_2_ section relieved more stored internal energy, causing results to be away from the target values.

**Figure 11:**
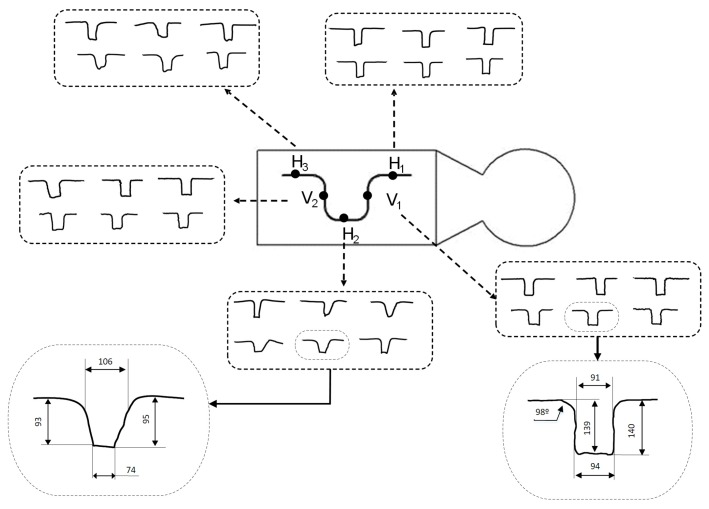
Microchannel shape analysis.

Finally, the microchannel shapes are presented in Figure 11. Two sets of specimens of different combinations of process parameters are shown for each microchannel section and three replicates for each combination. It can be seen that there are no differences in microchannel shape between the replicates of each combination, even when different sets of different combinations of process parameters are compared. This reinforces that velocity and amplitude are not significant to microchannel shape.

The shape of sections H_1_, V_1_, and V_2_ are similar and mostly well defined in both the walls and the bottom. On average, the difference of microchannel width between the surface and the top is around 11 µm, the difference in depth is 7 µm, and the radius of the top is around 98°. In contrast, the worst resolution is observed for section H_2_. In that case, deviations are around 45 µm in width and 15 µm in depth. Here, the shape is not well defined and the walls are not parallel to each other. In the case of H_3_, the shape of the microchannel is between those of H_1_ and H_2_. 

## 4. Conclusions

In this research, thin-wall plates with polystyrene microchannels were successfully manufactured by ultrasonic molding technology. A rigorous statistical procedure was followed using a large set of experimental data that were analyzed to demonstrate the repeatability and replicability of this technology.

The results reveal that plunger velocity affects both transparency and filling cavity. Higher velocity improves transparency but decreases cavity filling, so intermediate values are required to guarantee both requirements. Overlapping the corresponding response surfaces, the optimal values can be selected. The vibration amplitude has slightly less influence in both cases. Degradation symptoms appear when the material receives too much ultrasonic energy, coming from high levels of amplitude and low plunger velocity. 

The average deviation of part thickness is less than 7%, but the research shows that cooling time becomes a relevant factor to assure this property. The principal component analysis (PCA) method becomes very useful to evaluate the thickness data and analyze them regarding the mold dimensions. 

Replication accuracy of the microchannel is better in depth than in width, obtaining an average deviation of 4% and 11%, respectively. This replication also depends on the orientation of the microchannels, so that the results of cross-flow sections are little more dispersed than in-flow ones. The distance from the injection entrance also has an influence on these results. 

In further research, a more complex microchannel geometry should be tested to guarantee the feasibility of this technology for processing microfluidic devices. In that sense, other polymers should be studied, like cyclic olefin copolymer (COC) and polymethyl methacrylate (PMMA). Regarding the process parameters, the influence of cooling time and compaction force should be studied. S

## Figures and Tables

**Table 1:** Mold characterization results.

		Cavity Thickness				Microchannel			
		Mean (µm)	Error (%)			Depth		Width	
						Mean (µm)	Error (%)	Mean (µm)	Error (%)
**Corner**	T_1 Mold_	566	13	**Microchannel** **Section**	H_1 Mold_	137	8.7	73	7
	T_2 Mold_	576	15.2		V_1 Mold_	151	0.7	71	9
	T_3 Mold_	580	16		H_2 Mold_	139	7.3	70	10
	T_4 Mold_	565	13		V_2 Mold_	136	9.3	70	10
					H_3 Mold_	139	7.3	76	4
	**Avg.**		14.35		**Avg.**		6.67		8

**Table 2:** Part transparency classification.

	Level	Qualitative Description	Transmittance Value (%)	Transparency Level (Examples)
**Blurred**	1	76–100% of cloudy zones	<85	Level 1 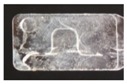
	2	51–75% of cloudy zones		
	3	26–50% of cloudy zones	87.366	Level 3 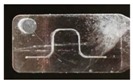
	4	1–25% of cloudy zones		
**Transparent**	5	Transparent with some defects	93.436	Level 6 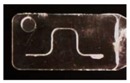
	6	Transparent with isolated defects	94.043	
	7	Transparent without defects	98.647	

**Table 3:** Summary of the experimental results; k = 2.

	Velocity (mm/s)	Amplitude (μm)	Filling (%)	Transparency (Qualitative Scale)	Thickness Dimensions (Standard Deviation) (μm)			
					*T_1Part_*	*T_2Part_*	*T_3Part_*	*T_4Part_*
**Phase 1: 2^K^ experiment + central point**	1	14.06	100	5.3	590 (±13)	583 (±9)	582 (±4)	586 (±7)
			100	5.4	583 (±8)	582 (±6)	579 (±10)	575 (±6)
			100	5.7	585 (±11)	578 (±12)	581 (±10)	586 (±13)
		70.31	100	6	590 (±5)	586 (±4)	585 (±7)	586 (±5)
			100	5.9	587 (±6)	583 (±12)	578 (±7)	579 (±10)
			100	5.8	582 (±12)	578 (±10)	577 (±6)	579 (±9)
	4	14.06	46	7	–	–	–	–
			80.50	5.50	566 (±3)	565 (±7)	571 (±12)	561 (±8)
			83.33	5.67	582 (±10)	569 (±1)	576 (±6)	574 (±12)
		70.31	62	7	–	–	–	–
			65.50	6.4	–	–	–	–
			70	6.2	585 (±2)	562 (±3)	571 (±5)	563 (±8)
	2.5	42.19	87.50	6.6	582 (±3)	573 (±1)	576 (±4)	577 (±6)
			85	6.7	569 (±7)	561 (±9)	577 (±9)	571 (±8)
			81	6.7	567 (±8)	562 (±11)	565 (±11)	566 (±4)
**Phase 2: 3^K^ experiment**	1	42.19	100	6	576 (±7)	577 (±6)	570 (±9)	571 (±9)
			100	5.7	592 (±9)	594 (±4)	603 (±9)	586 (±8)
			100	6	583 (±12)	592 (±7)	590 (±13)	581 (±8)
	4	42.19	45	7	–	–	–	–
			42.50	7	–	–	–	–
			41	6.7	–	–	–	–
	2.5	70.31	82	6.6	565 (±5)	565 (±3)	564 (±4)	555 (±13)
			90	5.78	–	–	–	–
			82	6.75	–	–	–	–
		14.06	98	6	572 (±17)	577 (±9)	575 (±10)	575 (±7)
			94	6	–	–	–	–
			99	5	578 (±9)	577 (±16)	572 (±14)	576 (±16)
